# Combinatorial administration of insulin and vitamin C alleviates the cerebral vasospasm after experimental subarachnoid hemorrhage in rabbit

**DOI:** 10.1186/1471-2202-12-77

**Published:** 2011-08-01

**Authors:** Shouchun Li, Jinghui Xue, Jixin Shi, Hongxia Yin, Zhiwen Zhang

**Affiliations:** 1 Department of Neurosurgery, First Affiliated Hospital of Chinese PLA General Hospital, Beijing, PR China; 2 Department of Neurosurgery, General Hospital of Nanjing Military Region, Nanjing, PR China

## Abstract

**Background:**

Cerebral vasospasm (CVS) is a common serious complication after the spontaneous subarachnoid hemorrhage (SAH). Despite recent advances in medical and surgical treatments, the 30-day mortality rate of SAH remains high, and there is lack of especially effective clinical treatment to alleviate and improve CVS. The present study has investigated the therapeutic effect of insulin and vitamin C on CVS after SAH.

**Results:**

Five days after SAH, there is obvious basilar artery spasm in SAH group, whose average vascular cross-sectional area (233,099 ± 16,750 μm^2^) is significantly smaller than that in control group (462,128 ± 74,756 μm^2^), which is also significantly different from those in SAH + insulin group (221,114 ± 43,457 μm^2^) and SAH + vitamin C group (237,820 ± 21,703 μm^2^). SAH + insulin + vitamin C group shows no evident vasospasm and maintains a vascular cross-sectional area of 425,530 ± 45,503 μm^2^, which is significantly different from that in SAH group. Insulin receptor α (InRα) expression is significantly downregulated in the vascular endothelial cells of SAH, SAH + insulin, and SAH + vitamin C groups (*P *< 0.01) but remains unchanged in vascular endothelial cells of SAH + insulin + vitamin C group (*P *> 0.05). Five days after SAH, serum and cerebrospinal fluid NO levels in SAH, SAH + insulin, and SAH + vitamin C groups decrease significantly (*P *< 0.01) compared to that in control group, whereas the reduction is not evident in SAH + insulin + vitamin C group (*P *> 0.05).

**Conclusion:**

Combinatorial treatment with insulin and vitamin C has effectively relieved the CVS after SAH in rabbit, possibly through increasing the InRα expression and NO level, whereas treatment with insulin or vitamin C alone fails to do so.

## Background

CVS is a common serious complication after the spontaneous SAH. It induces and aggravates cerebral ischemic injury and becomes one of the major causes of disability and mortality after aneurysm rupture. Although some medications like non-selective calcium blockers can be administered, there is still lack of especially effective clinical treatment to alleviate and improve CVS [1]. How to prevent and improve CVS is an urgent problem facing the neurosurgeons. Studies have confirmed that in addition to regulating metabolism, promoting cell growth and proliferation, and inhibiting apoptosis, insulin also plays a strong vasoactive role, dilates the vessels, increases blood flow, and improves metabolism in tissues [2]. The vasodilatory effect of insulin is achieved through increasing the synthesis and release of NO in vascular endothelial cells [3]. The post-SAH oxidation of oxyhemoglobin into methemoglobin and the release of large amount of oxygen free radicals cause serious injury in endothelial cells, which becomes one of the major mechanisms underlying the occurrence of CVS and the brain damage. Although vitamin C cannot directly dilate the blood vessels, it has powerful antioxidant effect to remove and inhibit oxygen free radicals and to improve the function of endothelial cells [4], thus may help insulin in regulating blood vessels. Therefore, this study has investigated the therapeutic effect of combinatorial use of insulin and vitamin C in treatment of post-SAH CVS in a rabbit model to provide experimental basis for future clinical treatment.

## Results

### The cross-sectional area of basilar artery

The vascular cross-sectional area in SAH + insulin + vitamin C group is 425,529.9 ± 45,502.98 μm^2^, which shows no significant difference from that in control group (462,127.9 ± 74,755.82 μm^2^, *P *= 0.605). The vascular cross-sectional areas in SAH, SAH + insulin, and SAH + vitamin C groups are 233,099.2 ± 16,750.12 μm^2^, 221,113.6 ± 43,456.86 μm^2^, and 237,819.6 ± 21,703.01 μm^2^, respectively, and all are significantly different from that in control group (*P *< 0.01). There is significant difference between SAH + insulin + vitamin C group and SAH group (*P *< 0.01, Table 1).

**Table 1:** Cross-sectional area of basilar artery ( ± **S E**) and InRα expression on the endothelial cells

			InRα expression			Rank difference
				
Groups	n	Cross-sectional area (μm^2^)	0	1	2	
Control	5	462127.9 ± 74755.82	0	0	5	
SAH	10	233099.2 ± 16750.12*	8	2	0	24.80*
SAH + Vc	10	237819.6 ± 21703.01*	7	2	1	22.00*
SAH + In	10	221113.6 ± 43456.86*	6	4	0	21.60*
SAH + In + Vc	10	425529.9 ± 45502.98	0	3	7	3.60

In: insulin; Vc: vitamin C; *: *P *< 0.01 compared to control group; ^Δ^: *P *< 0.01 compared to SAH + In + Vc group; InRα expression: 0 (negative), 1 (positive), and 2 (strongly positive).

### Immunohistochemistry

The expressions of InRα in basilar artery endothelial cells in SAH, SAH + insulin, and SAH + vitamin C groups are significantly lower than that in control group (*P *< 0.01), whereas the InRα expression in endothelial cells in SAH + insulin + vitamin C group has been significantly increased and different from those in SAH + insulin and SAH + vitamin C groups (*P *< 0.01, Table 1).

### Hematoxylin and eosin (H&E) staining

**Figure 1:**
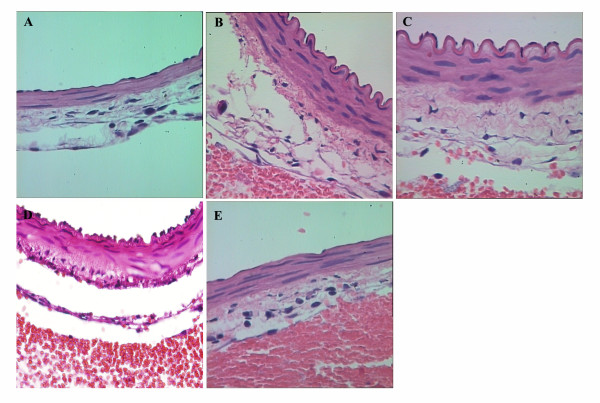
**H&E staining of basilar artery (Magnification: × 400)**. A (control group) shows spreading of endothelial cells and no thickening of the vessel wall; B (SAH group) shows significant thickening of vessel wall and shrinkage of endothelial cells, manifesting battlement-like morphological changes; C (SAH + insulin group) exhibits significant vessel wall thickening and endothelial cell shrinkage with battlement-like changes too; D (SAH + vitamin C group) shows significant wall thickening and endothelial cell shrinkage, manifesting battlement-like changes; E (SAH + insulin + vitamin C group) shows spreading of endothelial cells with moderate wall thickening.

Staining of the sections from SAH group shows significant thickening of the basilar artery wall, shrinkage, detachment, and falling off of the endothelial cells, manifesting typical battlement-like changes (Figure 1B). SAH + insulin or SAH + vitamin C group shows evident vessel wall thickening, battlement-like change, endothelial cell shrinkage, and detachment (Figure 1C and 1D), whereas combinatorial treatment group exhibits mild thickening of the vessel wall and stretch of the endothelial cells with rare or no detachment (Figure 1E).

### NO content

**Figure 2:**
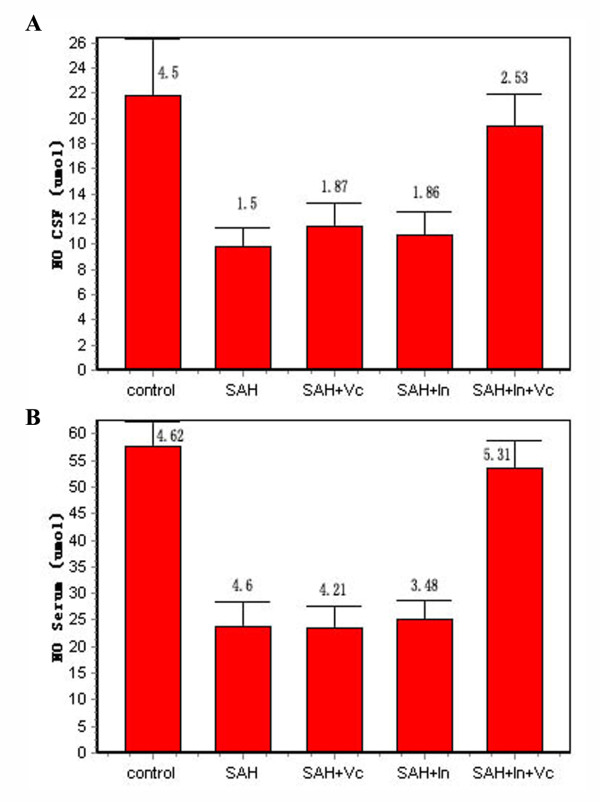
**NO contents in cerebrospinal fluid and serum**. A shows NO contents in cerebrospinal fluid, and B shows NO contents in serum.

In the fifth day after SAH, NO levels in serum and cerebrospinal fluid decrease significantly (*P *< 0.01). After combinatorial treatment with insulin and vitamin C, NO level increases significantly in serum and cerebrospinal fluid (*P *< 0.05), whereas insulin or vitamin C alone fails to increase the NO level (*P *< 0.01, Figure 2).

### Glucose and insulin contents

Serum glucose level in the animals shows significant transient increase at 30 min after the blood injection, which is significantly higher than the normal reference value (*P *< 0.05), but exhibits no significant changes at other times after the injection. Although serum insulin increases at 30 min after the blood injection and remains at a higher level, the difference is not significant when compared to the original level before the blood injection; however, the differences become significant at the third and fifth days after the injection (*P *< 0.05, Table 2).

**Table 2:** Blood glucose and insulin levels ( ± SE) before and after blood injection

Time	Blood glucose (mmol/L)	Insulin (IU)
Before injection	6.13 ± 0.65	13.46 ± 3.80
30 min	10.64 ± 2.83*	37.38 ± 9.57
60 min	7.64 ± 2.91	42.65 ± 9.60
90 min	6.36 ± 0.63	43.94 ± 12.37
SAH1D	6.72 ± 0.30	40.43 ± 7.79
SAH3D	7.75 ± 1.63	51.35 ± 16.83*
SAH5D	7.51 ± 2.12	67.15 ± 16.36*

30 min, 60 min, 90 min, SAH1D, SAH3D, and SAH5D: the times after blood injection; *: *P *< 0.05 compared to control.

## Discussion

In this study, combinatorial use of insulin and vitamin C has successfully alleviated the CVS after SAH, though individual administration of either insulin or vitamin C fails to achieve the desired effects, which confirms the destructive effect of oxygen free radicals on vascular endothelial cells and the significant vasodilatory effect of insulin. These results are consistent with those from Hirashima *et al*. [4].

Insulin resistance is the state of tissues and organs with reduced sensitivity to insulin. Previous studies have confirmed that inhibition of insulin's vasoactive effect induces insulin resistance and hyperinsulinemia, which is the major cause of atherosclerosis and coronary artery spasm [5-7]. The present study has demonstrated that one day after blood injection, the cross-sectional area of basilar artery in experimental animals decreases significantly with apparent constriction of the blood vessels, which becomes mostly significant at the third day post blood injection (that is the fifth day of the study), relieves then gradually, and recovers at the fifth day. Although the glucose level only increases within 30 min after the blood injection, the plasma insulin level begins to rise continuously 30 min after the injection and reaches its maximum 5 days after the second injection, implying that CVS is associated with hyperinsulinemia. Experiments have confirmed that sustained high insulin level may promote the pathological changes of blood vessels, which forms an independent risk factor for vascular diseases. Firstly, hyperinsulinemia itself may downregulate insulin receptor by reducing the number and expression of insulin receptor in vascular endothelial cells, resulting in insulin resistance [8]; secondly, hyperinsulinemia activates protein kinase C (PKC) and abnormally increases its activity; thirdly, hyperinsulinemia promotes the proliferation of vascular smooth muscle cells and the synthesis and secretion of endothelin-1 (ET-1) in endothelial cells [9]. These delayed pathophysiological changes are also characteristic in CVS after SAH [1,10].

Therefore, according to the results of this study, it is reasonable to speculate that insulin resistance also exists in the present model of CVS after SAH. The number and expression of insulin receptor on the membrane of endothelial cells in the basilar artery with severe spasm have been significantly reduced, whereas the expression of insulin receptor on the endothelial cells of basilar artery without spasm is strongly positive. Downregulation of the number and expression of insulin receptor causes insulin resistance and inhibition of the various physiological effects of insulin, including its vasoactive effect [8]. Vascular endothelial cells are the target cells for insulin to exert its vascular regulatory effects. Extensive studies have demonstrated that there are a large number of insulin receptors on vascular endothelial cells; upon binding with insulin, the downstream signaling pathways are activated to further stimulate the production of ET-1 and NO in endothelial cells, adjust the balance of vascular constriction, and hence exert the vasoactive effect [11]. However, as shown in this study, the vascular endothelial cells are damaged after SAH; the number and expression of insulin receptor on these cells are reduced significantly, resulting in insufficient binding with insulin and failure in promoting the release of the vasodilatory factor NO, which hampers the vasoactive effect of insulin. Therefore, treatment with insulin alone fails to alleviate the CVS, whereas combinatorial use of insulin and vitamin C effectively relieve the symptom. Vitamin C is an antioxidant; although itself has no direct role on the vasomotor and hemodynamics [4], vitamin C can counteract the damaging effect of oxygen free radicals on the endothelial cells, improve cell function, reduce insulin resistance, increase the sensitivity to insulin in endothelial cells, promote the binding of insulin with its receptor, and thereby increase the release of vasodilatory factor NO to exert its vasoactive effect. In addition, vitamin C can inhibit the activity of oxygen free radicals and inhibit their binding to NO, thus facilitating the vasodilatory effect of NO [12,13]. Therefore, combinatorial use of insulin and vitamin C has significantly improved CVS in animals of SAH + insulin + vitamin C group.

However, the most serious complication of insulin therapy is low blood glucose reaction. In this study, the blood glucose has been controlled at a level higher than 4.9 mmol/L (the normal range of blood glucose level in rabbit is 4.33 ~ 8.60 mmol/L), and there is no occurrence of twitch, coma, and other symptoms in the animals due to low blood glucose level.

## Conclusions

The present study has successfully alleviated the post-SAH CVS in rabbit through combinatorial treatment with insulin and vitamin C. However, since the cerebral vascular structures in human are significantly different from that in rabbit, the effect of this method in treating CVS in human needs further investigations.

## Methods

The animal protocols in this study were approved by the institutional Laboratory Animal Ethics Committee; all the animals were treated humanely in accordance with the guidelines for the Care and Use of laboratory Animals published by the U.S. National Institutions of Health (NIH Publication No. 85-23, revised 1996).

### Animals and the establishment of SAH model

Total 65 male New Zealand white rabbits, weighing 2.8 ~ 3.1 kg, were purchased from Experimental Animal Center of Jiangsu Province. Animals were rested for one week in the animal laboratory of our hospital. Post-SAH basilar artery spasm model was induced by two times of intracisternal injection of autologous blood [14]. Briefly, animals were anesthesized by intramuscular injection of ketamine (25 mg/Kg) and droperidol (1.0 mg/Kg), placed in lateral position, and covered with sterile surgical hole towel. Cisternal puncture was performed percutaneously with No. 7 lumbar puncture needle. After drainage of 1 mL of cerebrospinal fluid, 1.5 mL fresh blood were drawn rapidly from the central artery in rabbit ear and injected into the cistern magna within 20 seconds. After completing the blood injection, animals were placed rapidly in head-low position at 30°C for about 5 min to facilitate the blood dispersing in the brain. The same procedure was repeated after 48 h. The rectal temperature of animal was maintained at 37°C with heated blankets.

### The grouping and treatment of animals

Total 45 animals were randomly divided into 5 groups: 1) control group (n = 5) was injected with 1.5 mL saline through cisternal puncture; 2) SAH group (n = 10) received no treatment after the blood injection; 3) SAH + insulin group (n = 10) was treated by subcutaneous injection of insulin at 0.2 U/kg, starting 30 min after the blood injection, 3 times/d; 4) SAH + vitamin C group (n = 10) was treated by intramuscular injection of vitamin C at 40 mg/kg, starting 30 min after the blood injection, 3 times/d; 5) SAH + insulin + vitamin C group (n = 10) was treated by subcutaneous injection of insulin at 0.2 U/kg plus intramuscular injection of vitamin C at 40 mg/kg, starting 30 min after the blood injection, 3 times/d. During the process of model induction, groups with rabbits died from rapid increase in intracranial pressure due to the blood injection were supplemented randomly. The glucose level in ear blood and the behavior of animals in insulin treated groups were monitored 2 h after insulin injection. All the animals were killed at the fifth day after blood injection by perfusion. Another 20 rabbits were randomly divided into the following four groups: 1) control group (n = 5) was injected intracisternally with 1.5 mL saline; 2) SAH1D group (n = 5) was killed one day after the blood injection; 3) SAH3D group (n = 5) was killed three days after the blood injection; 4) SAH5D group (n = 5) was used for the measurement of blood glucose and insulin levels; the blood drawn from marginal vein of rabbit ear was tested for glucose (with rapid glucometer) and insulin contents respectively at 10 min before the puncture, at 30, 60, and 90 min after the blood injection, as well as at 1 h before each meal on the first, third, and fifth days after the injection.

### The perfusion

All animals were anasthesized by intramuscular injection of ketamine (25 mg/kg) and droperidol (1.0 mg/kg), placed in supine position, and fixed on the operating table. Perfusion was carried out after drawing 2 mL blood from ear vein and 2 mL cerebrospinal fluid from each animal [15]. Briefly, 37°C neutral phosphate buffer was firstly injected into the body through left ventricle and replaced by 37°C 10% neutral formalin to fix the tissues immediately after the effluence was in light color. After perfusion, the brain tissues with basilar artery were preserved in 10% neutral formalin and sent to the Department of Pathology for further processing.

### Specimen preparation and determination of the arterial diameter

Samples were collected from the midpoint of proximal, middle, and distal portions of the basilar artery respectively, embedded, and cut into five consecutive 4 μm sections by the staff in Department of Pathology, who was double-blind for both the experimental design and grouping. Sections were stained with H&E, and photographs at 4 × magnification were collected with high-resolution medical image analysis system (HMIAP, 2000. Tongji Medical University, China). The inner perimeters (L) of basilar arteries in each section were measured with ImageJ software package (USA), and the vascular cross-sectional area was calculated by Excel software according to the formula S (cross-sectional area) = πL^2^/4.

### The measurement of blood glucose and insulin

Blood glucose levels were measured with fast glucometer; serum insulin levels were determined by radioimmunoassay method with Insulin RIA kit (Beijing FuRui Biosciences, Beijing, China) following the manufacturer's instructions.

### Detection of NO

NO content was determined indirectly by colorimetry method. Briefly, on the fifth day of SAH induction, 2 mL of ear venous blood and 2 mL of cerebrospinal fluid were collected and centrifuged at 3000 rpm, and the supernatants were harvested for further NO detection with the special kit provided by the Academy of Military Medical Sciences (Beijing, China) following the manufacturer's instructions.

### Immunohistochemical staining

Immunohistochemical staining with mouse anti-insulin receptor α antibody (Bioscience Company, USA) and evaluation of insulin receptor α expression in basilar artery endothelial cells were done by staff from the Department of Pathology (under double-blind condition). The expression of insulin receptor α was classified into three levels as the following: 0 (negative), 1 (positive), and 2 (strongly positive).

### Statistics

The data of cross-sectional area, blood glucose, insulin, and NO content were presented as mean ± standard error ( ± SE) and analyzed with SPSS13.0 software package (one-way ANOVA and followed by student t-test). Endothelial expression of InRα among different groups was analyzed using Mann-Whitney test. Differences with *P *< 0.05 were considered as significant.

## Abbreviations

CVS: cerebral vasospasm; SAH: subarachnoid hemorrhage.

## Competing interests

The authors declare that they have no competing interests.

## Authors' contributions

YHX bred the rabbits and participated in making experiment animal model. XJH performed Immunohistochemical staining. SJX and ZZW participated in overall design of the study and the writing of the manuscript. All authors read and approved the final manuscript.
